# Potentially inappropriate medication among older patients with diabetic kidney disease

**DOI:** 10.3389/fphar.2023.1098465

**Published:** 2023-02-08

**Authors:** Yuping Wang, Jie Zhu, Luchen Shan, Ling Wu, Cunchuan Wang, Wah Yang

**Affiliations:** ^1^ Department of Pharmacy, The First Affiliated Hospital of Jinan University, Guangzhou, China; ^2^ Department of Metabolic and Bariatric Surgery, The First Affiliated Hospital of Jinan University, Guangzhou, China; ^3^ Guangdong-Hong Kong-Macao Joint University Laboratory of Metabolic and Molecular Medicine, Guangzhou, China; ^4^ Department of Gastrointestinal Surgery, The Second People’s Hospital of Yibin, Yibin, Sichuan, China; ^5^ College of Pharmacy, Jinan University, Guangzhou, China

**Keywords:** potentially inappropriate medication, polypharmacy, diabetic kidney disease, hospitalised and dicharge patient, older patients

## Abstract

**Objective:** Potentially inappropriate medications (PIM) contribute to poor outcomes in older patients, making it a widespread health problem. The study explored the occurrence and risk factors of PIM in older diabetic kidney disease (DKD) patients during hospitalization and investigated whether polypharmacy was associated with it.

**Methods:** Retrospective analysis of the patients ≥ 65 years old diagnosed with DKD from July to December 2020; the PIM was evaluated according to the American Beers Criteria (2019). Factors with statistical significance in univariate analysis were included in Logistic multivariate analysis to explore the potential risk factors related to PIM.

**Results:** Included 186 patients, 65.6% of patients had PIM, and 300 items were confirmed. The highest incidence of PIM was 41.7% for drugs that should be carefully used by the older, followed by 35.3% that should be avoided during hospitalization. The incidence of PIM related to diseases or symptoms, drug interactions to avoid, and drugs to avoid or reduce dose for renal insufficiency patients were 6.3%, 4.0% and 12.7%, respectively. The medications with a high incidence of PIM were diuretics (35.0%), benzodiazepines (10.7%) and peripheral ɑ1 blockers (8.7%). Compared with hospitalization, there were 26% of patients had increased PIM at discharge. Multivariate Logistic regression analysis showed that polypharmacy during hospitalization was an independent risk factor for PIM, OR = 4.471 (95% CI: 2.378, 8.406).

**Conclusion:** The incidence of PIM in hospitalized older DKD patients is high; we should pay more attention to the problem of polypharmacy in these patients. Pharmacists identifying the subtypes and risk factors for PIM may facilitate risk reduction for older DKD patients.

## 1 Introduction

More than 400 million people are suffering from diabetes mellitus (DM) around the world, and nearly half of them are older people (≥65 years old) (Bellary et al., 2021). About 20%–40% of patients with diabetes would develop into diabetic kidney disease (DKD) (Afkarian et al., 2016); it is associated with an increased risk of adverse health outcomes, impaired quality of life, and premature mortality (Alicic et al., 2017).

Older diabetes is a complex and heterogeneous group; in addition to diabetes and associated complications, these patients are still at an increased risk of geriatric syndromes, including falls, chronic pain, depression, and functional and cognitive decline (Clemens et al., 2019). Because of various diseases, older people with DKD have an increased risk of taking multiple medications. A study from Germany showed that 70.4% of patients with an eGFR<60 mL/min took at least five medications, and 17.7% of them took ≥10 medications for a long time (Dorks et al., 2016). But polypharmacy therapy (Typically defined as using five or more pharmaceuticals simultaneously) is related to adverse drug reactions (ADR), common and possibly preventable causes of accidental hospitalization, increased morbidity, mortality and medical care costs. The risk for ADR in patients taking two medications at the same time was 13%, while the risk for patients taking four, seven or more were 38% and 82%, respectively (Zazzara et al., 2021). Therefore, it is necessary to identify and avoid PIM for older patients with DKD.

Many tools can be used to identify the PIM in multiple medications prescription. The American Geriatrics Society Beers Criteria (AGS Beers Criteria) has been proven to be more sensitive than other tools in reducing drug-related adverse events, emergency visits and hospitalization, improving the overall health of patients (Brown et al., 2016). It can find the prevalence of PIM among older patients with different diseases and figure out the types of medications involved in it.

The common PIM in old American drivers were taking medications known to damage driving ability and increase collision risks. The most common PIM treatment for them was benzodiazepines (accounting for 16.6% of total PIM), followed by non-benzodiazepines hypnotics (15.2%) and antidepressants (11.5%) (Li et al., 2019). A French study using this criterion showed that 64.8% of older patients with chronic diseases and multiple medications have PIM at least once (Guillot et al., 2020). In comparison, the prevalence of PIM among these patients of hospitalized in China was higher (about 72.5%) (Tian et al., 2021a). The common medications induced PIM in patients with the chronic coronary syndrome in Beijing were diuretics (37.1%), benzodiazepines (15.2%) and glimepiride (13.1%) after discharge (Zhao et al., 2021).

Polypharmacy is common in older DKD patients as they are more prone to problems such as synchronously controlling glucose, common comorbidities, elevated blood pressure and so on. Studies have reported that pharmacists may play a critical role in managing concurrent DKD, because of their unique perspectives on medications prescribed across conditions and providing actionable medication-related recommendations (Fazel et al., 2017; Zullig et al., 2020). A few studies have shown that the incidence of potentially inappropriate medication (PIM) is high in older patients with chronic kidney disease (CKD) (Roux-Marson et al., 2020; Luthke et al., 2022; Pehlivanli et al., 2022; Sharma et al., 2022); one has reported that proton pump inhibitors (PPI) were the most common medications induced PIM (Pehlivanli et al., 2022), while others revelled that polypharmacy and reduced estimated glomerular filtration rate (eGFR) were predictors for PIM in this population (Luthke et al., 2022; Sharma et al., 2022). There are studies on PIM in older diabetic patients (Oktora et al., 2021), but few studies have explored PIM in older diabetic nephropathy patients in China. The purpose of this study is: 1) To evaluate the PIM of these patients during hospitalization and discharge; 2) To Identify the risk factors of PIM as to provide a better reference for clinicians.

## 2 Materials and methods

### 2.1 Study design, setting, and patients

This retrospective study was carried out at the First Affiliated Hospital of Jinan University in China, a tertiary public general teaching hospital with 1900 beds. The study included patients aged 65 or older with DKD as the primary diagnosis, which was searched by the International Statistical Classification of Diseases and Related Health Problems (ICD-10) coding system for code N08 (DKD) (WHO International, 2020); those patients were admitted into hospital between July 2020 and December 2020. The anonymity and confidentiality of patients and their data were always protected.

**FIGURE 1:**
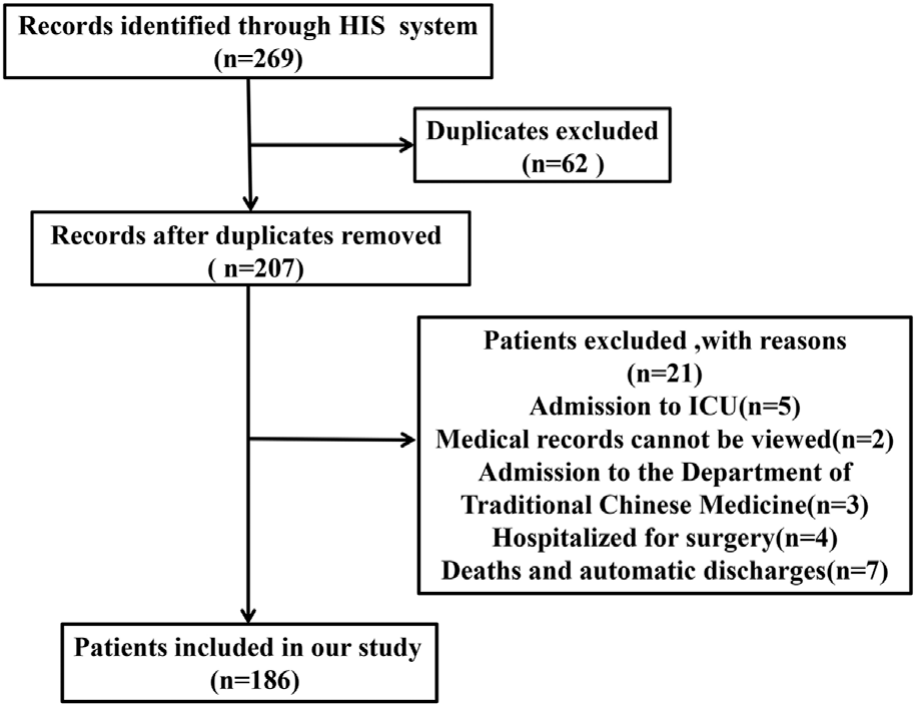
The flowchart of this study.

Total 269 medical records were extracted from patients aged 65 or older with DKD as the primary diagnosis through the hospital information system (HIS). The inclusion criteria were: 1) Patients aged 65 years or older and hospitalized for more than 24 h; 2) Patients who were not in the final stage of their disease undergoing palliative care; 3) Non-automatic discharge or death within 3 months; 4) Patients admitted to non-ICU or Chinese medicine department; 5) Admitted for non-surgical treatment. Exclusion criteria were: Patients hospitalized in the same department for two times or more within 3 months. The flow chart for selecting medical records is shown in (Figure 1).

### 2.2 Data collection

Two independent clinical pharmacists gathered data from the HIS; the following information was recorded: Age, sex, hospitalization ID, departments, the reasons for admission, length of stay, diagnosis, the number of complications and medications, the quantity of Chinese patent medicine, calculated Charlson Comorbidity Index (CCI) (to quantify the presence of co-existing diseases) and Creatinine Clearance (Ccr) [Cockcroft-Gault equation is used to estimate Cr clearance (Cockcroft and Gault, 1976)], both of them were used an online calculator.

### 2.3 Outcome measurements

According to the Beers Criteria (2019) (By the 2019 American Geriatrics Society Beers Criteria^®^ Update Expert Panel, 2019), drugs during hospitalization and discharge medication were evaluated (injection solvents and external preparations were excluded). The occurrence of PIM and drug types were analyzed: 1) Medications that should be avoided; 2) Medications that should be used with caution; 3) Medications with drug-disease/syndrome interactions; 4) Potentially clinically important drug-drug interactions to be avoided, with the severity of interactions was searched through Lexi-Interact (https://www.uptodate.com/drug-interactions); 5) Medications that should be adjusted along with kidney function. We referred to the designations of quality of evidence and strength of recommendations in Table 1 of the Beers Criteria (2019).

**TABLE 1:** Characteristics of the population and univariate analysis for PIM.

Variable	Patients with at least 1 PIM (*n* = 122)	Patients without PIM (*n* = 64)		P
Gender			X^2^ = 2.066	0.151
Male	65 (53.3%)	27 (42.2%)		
Female	57 (46.7%)	37 (57.8%)		
Age (year)			H = 0.622	0.430
65–74	64 (52.5%)	38 (59.4%)		
75–84	42 (34.4%)	18 (28.1%)		
≥ 85	16 (13.1%)	8 (12.5%)		
BMI	23.97 ± 3.52	23.64 ± 3.42	t = −0.626	0.532
Complicatoins				
Hypertension	109 (89.3%)	56 (87.5%)	X^2^ = 0.143	0.706
CHD	53 (43.3%)	28 (43.8%)	X^2^ = 0.002	0.968
Gout	34 (27.9%)	14 (21.9%)	X^2^ = 0.788	0.375
COPD	2 (1.6%)	1 (1.6%)	X^2^ = 0.002	0.968
SI	12 (9.8%)	5 (7.8%)	X^2^ = 0.207	0.649
HLP	20 (16.4%)	6 (9.4%)	X^2^ = 1,720	0.190
Reason for admission			X^2^ = 0.189	0.664
Infection	18 (14.8%)	11 (17.2%)		
Non-infection	104 (66.2%)	53 (82.8%)		
Department			H = 0.022	0.882
Nephrology	50 (41.0%)	27 (42.2%)		
Endocrinology	32 (26.2%)	18 (28.1%)		
Cardiology	12 (9.8%)	3 (4.7%)		
Others	28 (23.0%)	16 (25.0%)		
LOS			H = 4.164	0.041^*^
≤7 days	18 (14.8%)	17 (26.6%)		
8–14 days	70 (57.4%)	35 (54.7%)		
>14 days	34 (27.9%)	12 (18.8%)		
CKD stage			H = 1.278	0.258
Stage 1	5 (4.1%)	1 (1.6%)		
Stage 2	13 (10.7%)	8 (12.5%)		
Stage 3	37 (30.3%)	24 (37.5%)		
Stage 4	21 (17.2%)	13 (20.3%)		
Stage 5	46 (37.7%)	18 (28.1%)		
Dialysis			X^2^ = 1.576	0.209
Yes	33 (27.1%)	12 (18.8%)		
No	89 (72.9%)	52 (81.2%)		
CCI			H = 0.557	0.456
<5	47 (38.5%)	28 (43.8%)		
5–10	74 (60.7%)	36 (56.3%)		
>10	-	1 (0.8%)		
NOC			H = 4.827	0.028^*^
<7	32 (26.2%)	27 (45.2%)		
7–14	84 (68.9%)	35 (54.7%)		
>14	6 (4.9%)	2 (3.1%)		
NOD			H = 26.111	0.000^*^
1–5	1 (0.8%)	2 (3.1%)		
6–10	17 (13.9%)	30 (46.9%)		
11–20	96 (78.7%)	31 (48.4%)		
21–35	8 (6.6%)	1 (1.6%)		
TrMe	1.29 ± 1.09	1.14 ± 0.96	t = −0.907	0.365

PIM, potentially inappropriate medication; BMI, body mass index; CHD, coronary heart disease; COPD, chronic obstructive pulmonary disease; SI, systemic infections; HLP, hyperlipidemia; LOS, length of stay; CCI, charlson comorbidity index; NOC, numbers of complications; NOD, number of drugs; TrMe, Traditional medication; * With *p* < 0.05.

Two pharmacists manually identified PIM at the patient level; the senior pharmacist verified all PIM. All authors discussed any discrepancies until consensus was achieved. This study evaluated all medications listed in the patient records during hospitalization and discharge for PIM. The same medication was considered a different kind of PIM if found in the different tables of the 2019 Beers list of medications that should be separately counted.

### 2.4 Statistical analyses

Statistical analysis was performed using SPSS 26 (*SPSS Inc., Chicago, IL, USA*). Descriptive analysis was performed on the types of PIM and the involved drugs. The measurement data were expressed as Mean ± SD; the counting data were expressed as numbers and constituent ratios. Independent sample *t*-test was used for measurement data, chi-square test or Kruskal-Wallis test was used for classified variables, factors with statistical significance in univariate analysis were included in Logistic multivariate analysis to explore the potential risk factors related to PIM, with *p* < 0.05 was considered statistically significant.

## 3 Results

### 3.1 Baseline characteristics

A total of 186 patients were included in the study, including 92 males and 94 females, with an average age of 74.87 ± 7.44 years (65–93 years) and an average BMI of 23.87 ± 3.48 kg/m^2^. In addition to the diagnosis of DKD, the most common complications were hypertension, accounting for 88.7% (165/186), followed by coronary heart disease (43.5%) and gout or hyperuricemia (25.8%). Stage IV and V of CKD were 18.3% and 34.4%. Patients undergoing dialysis accounted for 24.2%, and 75.8% had no dialysis. The average length of stay was 11.8 ± 5.42 days. Patients using five more medications during hospitalization were 98.4%. And 65.6% of patients had at least one PIM during hospitalization, while 49.5% were at discharge. The demographic statistics are shown in Table 1. Patients using five more medications during hospitalization were 98.4%. The average number of medications were 13 (Interquartile range were 10–15) in hospitalization, while at discharge were 9 (Interquartile range were 6–12). And 65.6% patients had at least one PIM during hospitalization, while 49.5% at discharge (Table 2).

**TABLE 2:** Number of medications and PIM at admission and discharge.

	Admission n (%)	Discharge n (%)
Polypharmacy (>5)	183 (98.4)	146 (78.5)
Number of patients prescribed with PIM	122 (65.6)	92 (49.5)
1 PIM	48 (39.3)	47 (51.1)
2–3 PIM	57 (46.7)	38 (41.3)
4–6 PIM	17 (13.9)	7 (7.6)
Total number of PIM	300	188
Medications that should be avoided	106 (35.3)	62 (33.0)
Medications that should be used with caution	125 (41.7)	89 (47.3)
Potentially clinical important drug-drug interactions to be avoided	12 (4.0)	8 (4.3)
Medications with drug-disease/syndrome interactions	19 (6.3)	12 (6.4)
Medications that should be adjusted along with kidney function	38 (12.7)	17 (9.0)

PIM, potentially inappropriate medication; IQR, interquartile range.

### 3.2 Prescriptions with PIM for DKD patients

**FIGURE 2:**
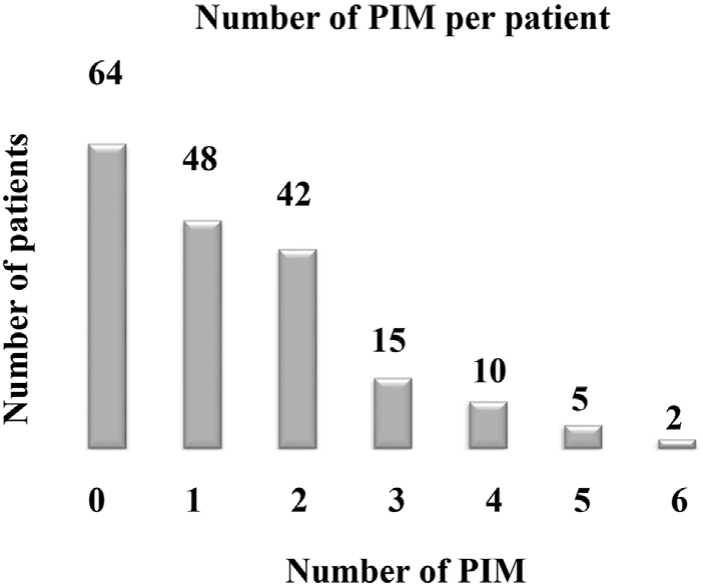
Number of potentially inappropriate medications per patient.

A total of 186 patients with DKD were included in the analysis, of which 122 patients (65.6%) had PIM. According to the criteria, patients having at least 1 item of PIM were 39.3%, while patients with 2-3items of PIM and 4-6 items were 46.7% and 13.9%, respectively (Figure 2; Table 2). Among the items included in the analysis, PIM occurred in 300 medication regiments during hospitalization, 106 (35.3%) items of drugs should be avoided, 125 (41.7%) should be used with caution, 38 (12.7%) were a dosage that should be adjusted according to renal function or to is avoided. In comparison, there were 62 (33.0%), 89 (47.3%), and 17 (9.0%) items, respectively, for discharge medications (Table 2). The medications with high incidence PIM were diuretics (35.0%), benzodiazepines (10.7%), peripheral ɑ1 blockers (8.7%) and spironolactone (6.7%) during hospitalization. In comparison, the medications at discharge were diuretics (35.6%), benzodiazepines (11.7%), peripheral ɑ1 blockers (10.6%) and novel oral anticoagulants (6.4%) (Table 3).

**TABLE 3:** Prescribed PIM according to the Beers criteria at admission and discharge.

The 2019 beers criteria	Quality of evidence^a^	Strength of recommendation^a^	PIM use frequency		
			Admission *n* = 300 (%)	Discharge *n* = 188 (%)	Change (%)
Medications that should be avoided			106 (35.3)	62 (33.0)	−2.3
Gastrointestinal medications	Moderate	Strong	2 (0.7)	0	−0.7
Antihistamines, first-generation	Moderate	Strong	2 (0.7)	0	−0.7
Digoxin>0.125 mg/d in heart failure	Moderate	Strong	1 (0.3)	1 (0.5)	0.2
Nifedipine, immediate release	High	Strong	10 (3.3)	1 (0.5)	−2.8
Sulfonylureas, long-acting	High	Strong	1 (0.3)	1 (0.5)	0.2
NSAIDs, non-selective	Moderate	Strong	10 (3.3)	3 (1.6)	−1.7
*Benzodiazepines*	*Moderate*	*Strong*	*32 (10.7)*	*22(11.7)*	*1.0*
Non-benzodiazepine	Moderate	Strong	10 (3.3)	5 (2.7)	−0.6
Barbiturates	High	Strong	1 (0.3)	1 (0.5)	0.2
Antipsychotic medications	Moderate	Strong	4 (1.3)	3 (1.6)	0.3
*Peripheral ɑ1 blockers*	*Moderate*	*Strong*	*26 (8.7)*	*20(10.6)*	*1.9*
Antidepressant medications	High	Strong	1 (0.3)	1 (0.5)	0.2
Endocrine agents	Moderate	Weak	5 (1.7)	3 (1.6)	−0.1
Antithrombotics	Moderate	Strong	1 (0.3)	1 (0.5)	0.2
Medications to be used with caution			125 (41.7)	89 (47.3)	5.6
SSRIs	Moderate	Strong	5 (1.7)	4 (2.1)	0.4
*NOAC-rivaroxaban and dabigatran*	*Moderate*	*Strong*	*7 (2.3)*	*12(6.4)*	*4.1*
*Diuretics*	*Moderate*	*Strong*	*105 (35.0)*	*67(35.6)*	*0.6*
Tramadol	Moderate	Strong	7 (2.3)	5 (2.7)	0.4
Aspirin for primary prevention	Moderate	Strong	1 (0.3)	1 (0.5)	0.2
Medications with drug-disease/syndrome interactions			19 (6.3)	12 (6.4)	0.1
Cardiovascular heart failure	High	Strong	7 (2.3)	4 (2.1)	−0.2
Parkinson disease	Moderate	Strong	3 (1.0)	2 (1.1)	0.1
History of fails or fractures	High	Strong	3 (1.0)	3 (1.6)	0.6
Kidney/urinary tract Chronic kidney disease stage 4 or higher	Moderate	Strong	5 (1.7)	3 (1.6)	−0.1
Gastrointestinal History of gastric	Moderate	Strong	1 (0.3)	0	−0.3
Medications that should be adjusted along with kidney function			38 (12.7)	17 (9.0)	−3.7
Spironolactone	Moderate	Strong	20 (6.7)	10 (5.3)	−1.4
Rivaroxaban	Moderate	Strong	4 (1.3)	2 (1.1)	−0.2
Enoxaparin	Moderate	Strong	4 (1.3)	0	−1.3
Famotidine	Moderate	Strong	2 (0.7)	1 (0.5)	−0.2
Pregabalin	Moderate	Strong	6 (2.0)	2 (1.1)	−0.9
Trimethoprim-sulfamethoxazole	Moderate	Strong	1 (0.3)	1 (0.5)	0.2
Colchicine	Moderate	Strong	1 (0.3)	1 (0.5)	0.2

Italic indicated the medications with high incidence of PIM.

PIM, potentially inappropriate medication; NSAIDs, Non-steroidal anti-inflammatory drugs; SSRIs, Selective serotonin re-uptake inhibitors; NOAC, novel oral anticoagulants.

^a^ Adapted from: By the American Geriatrics Society Beers Criteria Update Expert P, American Geriatrics Society 2019 Updated AGS, Beers Criteria (R) for Potentially Inappropriate Medication Use in Older Adults. J Am Geriatr Soc 67, 674–694 (2019).

**FIGURE 3:**
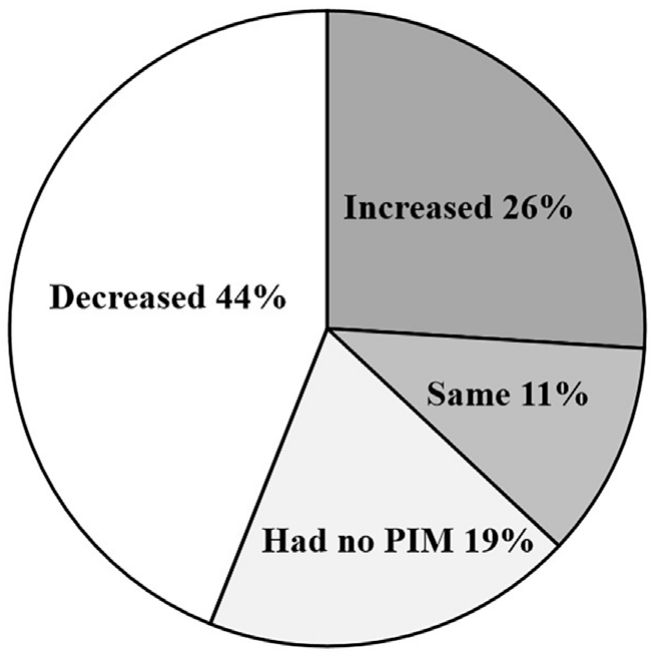
Change in the number of PIM between hospitalization and discharge. There were 44% patients had less potentially inappropriate medication (PIM) at discharge than hospitalization, 26% patients had more PIM than hospitalization at discharge; 19% patients had no PIM during hospitalization nor at discharge; 11% had the same PIM at release as in hospitalization.

According to the analysis of the changes in PIM between hospitalization and discharge medications, it was found that about 19% of patients had no PIM during hospitalization nor at discharge, 44% of patients had less PIM at discharge than hospitalization. But here a surprising thing was that 26% of patients still had more PIM than hospitalization at discharge, and 11% had the same PIM at release, which indicated that 37% of patients may still have drug-related adverse events due to PIM (Figure 3).

In this study, 12 cases of drug interactions that should be avoided, accounting for 4.0% of total PIM, including four issues of opioids and pregabalin (which may increase the risk of severe sedation-related adverse events including respiratory depression or death) and 1 case of opioids and benzodiazepines (may increase the risk of overdose). The above interactions were classified as level D in the *Up-to-date database*, which means the scheme needs to be modified. There were 3 cases of simultaneous use of potassium-preserving diuretics and RAS blockers. The drug interaction grade was C, and the risk of hyperkalemia should be monitored during simultaneous use. There were 4 cases of combination using ɑ1 blocker and a loop diuretic. The Beers Criteria suggested an increased risk of urinary incontinence in older women in this combination. Still, here was no specific interaction between the medications in the *Up-to-date database* (Table 4).

**TABLE 4:** Potentially clinical important drug-drug interactions to be avoided using the 2019 Beers Criteria.

Object drug/Class	Interacting drug/Class	Quality of evidence^a^	Strength of recommendation^a^	n (%)	Risk rationale	Severity
Potassium-sparing diuretics	RAS inhibitors	Moderate	Strong	3 (1.0)	Hyperkalemia or kidney injury	**C** ^**a**^
Opioids	Pregabalin	Moderate	Strong	4 (1.3)	Increased risk of severe sedation-related adverse events, including respiratory depression and death	**D** ^**b**^
Doxazosin	Furosemide	Moderate	Strong	4 (1.3)	Urinary incontinence	**NA** ^**c**^
Opioids	Benzodiazepines	Moderate	Strong	1 (0.3)	Increased risk of overdose	**D** ^**b**^

C^a^: monitor therapy; D^b^: consider therapy modification; NA^c^: no interaction.

RAS, Renin-angiotensin system.

^a^ Adapted from: By the American Geriatrics Society Beers Criteria Update Expert P, American Geriatrics Society 2019 Updated AGS, Beers Criteria(R) for Potentially Inappropriate Medication Use in Older Adults. J Am Geriatr Soc 67, 674–694 (2019).

### 3.3 Factors associated with PIM

**FIGURE 4:**
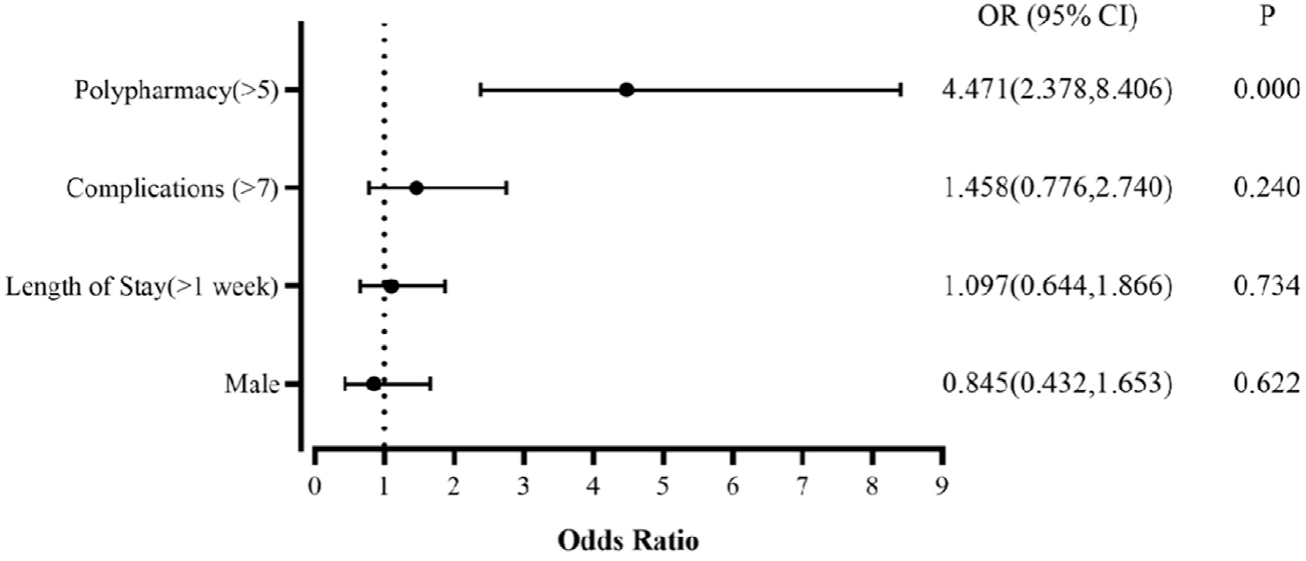
Binary logistics regression analysis of factors related to PIM.

Univariate analysis found that patients with longer hospitalization, more comorbidities and polypharmacy in hospitalization, were more likely to develop PIM (*p* < 0.05) (Table 1). Logistic multivariate analysis found that polypharmacy was an independent risk factor for PIM in older DKD patients, with an OR = 4.471 (95% CI:2.378, 8.406) (Figure 4).

## 4 Discussion

### 4.1 The incidence of PIM

For the first time, this study paid attention to PIM in older Chinese patients with DKD. It was found that 65.6% (122/186) patients had at least one PIM during hospitalization, while 49.5% were at discharge. Here a surprising thing was that 26% of patients still had more PIM than hospitalization at discharge.

The study has proved that there was little difference between the 2017 Chinese and 2019 AGS/Beers criteria (Tian et al., 2022), so the Beers criteria was also capable for Chinese. Our results are comparable to the PIM rate of (Beezer et al., 2022), who reported that the incidence of PIM was 61.1% during hospitalization in older patients with heart failure. Still, the PIM after discharge was higher, which was 64.0%. Our results are higher than those of Roux-Marson et al. (Roux-Marson et al., 2020), who found that 57.6% of older patients with advanced renal disease had PIM at least once. Although they included a cohort of older patients with age 75 years old or older, the drug categories 9 (7–11) are lower than this study 13 (4–35). The result was also higher than a single-center study in Japan, where patients with PIM at admission and discharge were 47.2% and 32.2%, respectively (Komagamine et al., 2019). However, people with lower eGFR often tend to have more PIM with more medications and are associated with a higher risk of readmission and death (Kang and Hong, 2019). Disparities in these results may be due to the different basis of the population included in studies, doctors’ distinctive prescription habits, and the accessibility of medications in different countries (Sarwar et al., 2017).

Compared with other studies (Al-Azayzih et al., 2019; Tian et al., 2021b), we also paid attention to the PIM of DKD patients’ discharged medications. Through our study, we suggested that pharmacists should pay more attention to the medication problem of patients with chronic diseases in the community, which is obviously a blank in China. We also found that pharmacists can identify the potential risks of DKD patients; they play a positive role in medication management and disease control for patients with chronic diseases. Generally speaking, for our study group, the incidence of PIM in older patients with DKD is higher, so it is more necessary for clinical pharmacists to participate in identifying PIM and medication monitoring. They can play a crucial role in medication reconciliation by minimizing the risk of PIM.

### 4.2 Related medications with PIM

The results showed that medications with high incidence of PIM in hospitalization were diuretics, benzodiazepines and peripheral α1 blockers, taking a percentage of 35.0%, 10.7%, and 8.7%, respectively. Diuretics were the most common medications with PIM, accounting for 35.0%, which was consistent with the incidence of PIM in patients with cardiovascular disease (Zahwe et al., 2019). In this study, 88.7% of patients were complicated with hypertension and 52.7% with stage IV-V renal insufficiency. Diuretics are often used to treat edema and hypertension, but excessive using will increase the risk of hypotension, renal impairment, and electrolyte abnormality. Based on the results, it is suggested that clinicians should closely monitor the electrolyte level and renal function during diuretics use in those populations.

Benzodiazepines (10.7%) are another common medication for PIM in hospitalization. Recent studies have found that 6%–9% of non-psychiatric department inpatients use antipsychotics, while 9%–12% of patients aged 65 or more use (Loh et al., 2014). A growing number of studies indicated that antipsychotics were widely used in hospitals for non-mental disease purposes, such as managing delirium or possible delirium (Herzig et al., 2016). High sensitivity to benzodiazepines in older people may lead to falls, cognitive impairment, delirium and dementia, increasing the risk of hip fractures in women (Diaz-Gutierrez et al., 2017). Therefore, psychological and behavioral therapy should be the first choice in treating insomnia in older people. If benzodiazepines cannot be avoided, short-acting and low-dose medications should be preferred. Meanwhile, medication education should be done, and monitoring of adverse reactions should be strengthened.

ɑ-blockers are usually used to control intractable or refractory hypertension in patients with CKD. Using ɑ-blockers in CKD patients is associated with a higher risk of kidney disease progression but a lower risk of cardiac events and mortality than alternative medications (Hundemer et al., 2021). Although it has a higher risk of postural hypotension, based on our study population (Patients with hypertension were 88.7%, but the proportion of refractory hypertension is unknown), whether to continue using should be weighed according to the patient’s situation.

The top three medications with high PIM at discharge were still diuretics (35.6%), benzodiazepines (11.7%) and peripheral ɑ1 blockers (10.6%). But what we should pay more attention to were novel oral anticoagulants (NOACs), accounting for 6.4%. In advanced CKD patients (i.e., Stage 4 and particularly stage 5), NOACs were not recommended due to the paucity of RCT data (Giugliano et al., 2013; Stamellou and Floege, 2018). Meanwhile, a major challenge of using anticoagulants among older patients was their higher risk of bleeding (Hasan et al., 2018). Importantly, patients receiving NOACs need regular checks of renal function to avoid overdosing. Therefore, patients at discharge should not only self-monitor the risk prescription of diuretics, benzodiazepines and peripheral ɑ1 blockers, but also pay attention to the bleeding or coagulation risk brought by the NOACs. Clinical pharmacists should strengthen relevant prescription education, which may be neglected.

In this study, the incidence of PIM in drug interactions that should be avoided was the lowest (4%). Although the proportion increased to 4.3% at discharge, the incidence was still lower than that reported by (Bories et al., 2021). However, our study mainly classified the drug interactions as C-D, indicating that about 4% of patients still need to monitor or adjust treatment regimens closely. Doctors prescribing medications for older patients need to understand the drug’s catabolic pathway, the protein binding rate, and the induction and inhibition of cytochrome P450 to avoid drug interactions caused by multiple medications. Drug-drug interaction prevention for ageing should be included in the drug monitoring plan.

An important finding in this study was that 26% of the included patients still had increased PIM at discharge. This indicates that about one-third of patients who did not experience PIM-related adverse events during hospitalization still face the potential risk of home medication due to the increased PIM after discharge. At the same time, PIM was significantly correlated with ADR in older patients with chronic diseases in China community (Li et al., 2015). Meanwhile, each patient used an average of 1.24 ± 1.04 kinds of Chinese patent medicine injections or oral preparations during hospitalization, among which oral practices will continue after discharge. Still, no relevant standards or guidelines regarding whether these medicines existed PIM. Wang et al. (2020) comprehensively analyzed the data in the Annual Report on Adverse Drug Reaction Monitoring from 2009 to 2018 released by the China National Medical Products Administration; they found that the proportion of ADR related to proprietary Chinese medicines was 10%–20%. The older people in China lack reliable knowledge of multiple medications (Lai et al., 2018). In China, pharmacists and family physicians rarely intervene in rational medication use at home for the older. How to guarantee and monitor the safety of home medication in the older is a problem worthy of further consideration by medical staff.

### 4.3 Factors associated with PIM

Although older DKD patients with longer hospitalization and more comorbidities were prone to have PIM in univariate analysis, there was no statistical significance in multivariate analysis. Multivariate analysis found that polypharmacy was an independent risk factor for PIM in these patients. Previous studies have shown that polypharmacy was significantly associated with PIM (Oliveira et al., 2012; Mazhar et al., 2018). Older patients often have multiple comorbidities; they are more susceptible to adverse effects of medications (Lees and Chan, 2011). Prescribing cascades occur when medications are treated for adverse effects of other existing medications, and as a result, older tend to accrue polypharmacy burden over time. Studies have been published that pharmacist-led deprescribing interventions were feasible and might lead to improved outcomes and cost savings for older with cancer and polypharmacy (Whitman et al., 2018) and that person-centered care provided by a multidisciplinary primary care team, including a pharmacist, can improve therapeutic adequacy in older patients (Rovira et al., 2022). Therefore, in our study the multidisciplinary team, including the pharmacist, was necessary to avoid or reduce PIM caused by polypharmacy in older DKD patients.

### 4.4 Strengths and limitations

This study has some limitations: 1) As a retrospective study, we only conducted a single-center study with a limited sample size on older patients with DKD, which might not represent the prevalence of PIM in all older with DKD due to different medication habits of doctors. 2) We only made PIM evaluations for hospitalization orders and discharge medications, excluding over-the-counter medications. However, Chinese patent medications, and dietary supplements may need to pay more attention to the actual PIM prevalence in this population. 3) We only analyzed PIM according to medical orders and could not assess potential prescription omission. Meanwhile, as the discharge medicine in China is limited to 7 days of course treatment, we could not evaluate medications with long-term use described in the Beers Criteria. Despite this, based on the consistency of current risk factors with previous reports, suggestions and references could be provided to implement corresponding medication intervention measures in the future.

## 5 Conclusion

The incidence of PIM in hospitalized older DKD patients is high; we should pay more attention to the problem of polypharmacy in these patients. Identifying the subtypes and risk factors for PIM may facilitate risk reduction for older DKD patients.

## Data Availability

The original contributions presented in the study are included in the article/supplementary material, further inquiries can be directed to the corresponding authors.
